# Interfacial Chemical Bridging Constructed by Multifunctional Lewis Acid for Carbon Nanotube/Silicon Heterojunction Solar Cells with an Efficiency Approaching 17.7%

**DOI:** 10.1002/advs.202206989

**Published:** 2023-02-23

**Authors:** Xian‐Gang Hu, Yi‐Ming Zhao, Hongyu Yang, Peng‐Xiang Hou, Chang Liu, Jingjing Chang, Yue Hao

**Affiliations:** ^1^ Advanced Interdisciplinary Research Center for Flexible Electronics Academy of Advanced Interdisciplinary Research Xidian University Xi'an 710071 China; ^2^ Shenyang National Laboratory for Materials Science Institute of Metal Research Chinese Academy of Sciences Shenyang 110016 China; ^3^ State Key Discipline Laboratory of Wide Band Gap Semiconductor Technology School of Microelectronics Xidian University Xi'an 710071 China

**Keywords:** carbon nanotubes, chemical bridges, heterojunction, stable doping, transparent electrodes

## Abstract

Single‐wall carbon nanotube/silicon (SWCNT/Si) heterojunction shows appealing potential for use in photovoltaic devices. However, the relatively low conductivity of SWCNT network and interfacial recombination of carriers have limited their photovoltaic performance. Herein, a multifunctional Lewis acid (p‐toluenesulfonic acid, TsOH) is used to significantly reduce the energy loss in SWCNT/Si solar cells. Owing to the charge transfer doping effect of TsOH, the conductivity and work function of SWCNT films are optimized and tuned. More importantly, a chemical bridge is constructed at the interface of SWCNT/Si heterojunction. Experimental studies indicate that the phenyl group of TsOH can interact with SWCNTs through *π*–*π* interaction, meanwhile, the oxygen in the sulfonic functional group of the TsOH molecule can graft on the dangling bonds of the Si surface. The chemical bridge structure effectively suppresses the recombination of photogenerated carriers. The TsOH coating also works as an antireflection layer, leading to a 19% increment of the photocurrent. As a result, a champion power conversion efficiency of 17.7% is achieved for the TsOH‐SWCNT/Si device, and it also exhibits an excellent stability, retaining more than 96% of the initial efficiency in the ambient air after 1 month.

## Graphical abstract

A multifunctional Lewis acid (p‐toluenesulfonic acid, TsOH) is used to significantly reduce the energy loss in single‐wall carbon nanotube/silicon (SWCNT/Si) solar cells. Owing to the charge transfer doping effect of TsOH, the conductivity and work function of SWCNT films are optimized and tuned. More importantly, a chemical bridge is constructed at the interface of SWCNT/Si heterojunction, which effectively suppresses the recombination of photogenerated carriers. As a result, a champion power conversion efficiency of 17.7% is achieved.

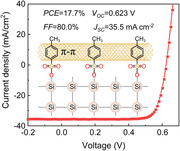

## 1 Introduction

The heterojunction formed by single‐wall carbon nanotube (SWCNT) films and n‐type silicon (Si) has attracted considerable interest in photovoltaic applications, such as solar cells and photodetectors, due to their simple structure and easy fabrication.^[^ ^1^, ^2^, ^3^, ^4^ ^]^ In the SWCNT/Si heterojunction, the SWCNT film usually acts as both transparent electrode and p‐type layer. Thus, the photovoltaic performances of SWCNT/Si heterojunction solar cells are affected by the transparency, sheet resistance, and work function of SWCNT films. Generally, the SWCNT transparent electrodes suffer from the intrinsic trade‐off between electrical conductivity and transparency as described by the Beer–Lambert law.^[^ ^5^ ^]^ It is important to further reduce the sheet resistance of the SWCNT films while keeping their high transparency. It is known that large bundle size and high contact resistance are two major issues resulting in the poor transparent conductive property of SWCNT networks.^[^ ^6^ ^]^ Large SWCNT bundle contributes little to the electrical conductivity but decreases the light transmission.^[^ ^7^ ^]^ As reported in our previous works,^[^ ^8^, ^9^, ^10^ ^]^ fabricating small‐bundle and isolated SWCNTs by optimizing preparation conditions of the floating catalyst chemical vapor deposition (FCCVD) technology is an effective strategy to achieve improved transmittance and conductivity for SWCNT electrodes. Moreover, surface charge transfer dopants, such as inorganic acids (HNO_3_, H_2_SO_4_, and HClSO_3_)^[^ ^11^, ^12^, ^13^, ^14^, ^15^ ^]^ and metal salts (AuCl_3_ and HAuCl_4_),^[^ ^16^, ^17^, ^18^, ^19^, ^20^ ^]^ are widely employed to further increase carrier concentration, reduce junction resistance, as well as tune the work function of SWCNT films. However, most of them were plagued by severe performance degradation over time and heating because of the weak absorption with SWCNTs or hygroscopic property in air ambient.^[^ ^21^ ^]^ Recently, the incorporation of organic acids (such as Nafion^[^ ^22^ ^]^ and polyacrylic acid^[^ ^23^ ^]^) into SWCNTs was found effective in obtaining both outstanding performance and long‐term stability.

The interface defect and poor contact at the SWCNT/Si heterojunction also limit the photovoltaic performance enhancement. Utilizing a thin passivation layer onto the Si surface could effectively reduce dangling bonds and surface trap states for Si‐based solar cells.^[^ ^24^, ^25^ ^]^ Materials such as hydrogenated amorphous silicon (a‐Si:H), ^[^ ^26^ ^]^ aluminum oxide (AlO_x_),^[^ ^27^ ^]^ and silicon oxide (SiO_x_)^[^ ^28^ ^]^ have been adopted to passivate the Si surface. However, these technologies require high vacuum equipment and complex preparation processes. Although the SiO_x_ layer could be formed on the Si surface by wet‐chemical oxidation agents,^[^ ^29^ ^]^ it is difficult to control its thickness because of the insulating property of SiO_x_. It was found that the optimum thickness of the SiO_x_ layer is ≈0.7 nm for pristine SWCNT/Si solar cells, and too thick or too thin thickness could not achieve a desirable photovoltaic performance.^[^ ^30^, ^31^ ^]^ Organic molecules with sulfonic groups can reduce the dangling bonds of the Si surface, resulting in an effective passivation effect.^[^ ^32^, ^33^, ^34^ ^]^ For example, Qian et al.^[^ ^22^ ^]^ and Chen et al.^[^ ^35^ ^]^ used Nafion to dope CNT films and to passivate the Si surface, as a result, SWCNT/Si devices with enhanced performance were obtained. Nevertheless, the interfacial structure and chemical bonding at the SWCNT/Si interface remain unclear.

In this study, we present a simple and efficient way to construct interfacial chemical bridge between SWCNT and Si by introducing a multifunctional Lewis acid (p‐toluenesulfonic acid, TsOH). It is found that the phenyl group of TsOH interacts with SWCNTs by *π*–*π* interplay, and the oxygen in the sulfonic group of the TsOH molecule grafts on the dangling bonds of the Si surface, resulting in a stronger interaction at the SWCNT/Si interface. In addition, the doping effect originated from the charge transfer between SWCNT and TsOH significantly improves the electrical conductivity and work function of SWCNT films. Meanwhile, the TsOH coating can serve as an antireflection layer in devices, causing a remarkable increase in photocurrent. Finally, a champion power conversion efficiency (PCE) of 17.7% with an open‐circuit voltage (*V*_OC_) of 0.623 V, a fill factor (FF) of 80.0%, and a short‐circuit current density (*J*_SC_) of 35.5 mA cm^−2^ was achieved for the TsOH‐SWCNT/Si solar cell, and it showed an excellent performance stability in air over 30 days.

## 2 Results and Discussion

**Figure 1:**
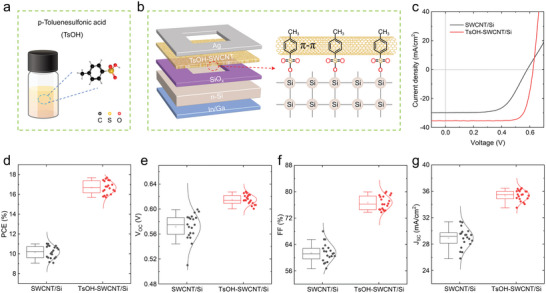
a) The p‐toluenesulfonic acid (TsOH) precursor solution and the molecular structure of TsOH. b) Device structure of the TsOH‐coated single‐wall carbon nanotube/silicon (TsOH‐SWCNT/Si) solar cell. c) Current density‐voltage (J‐V) curves, distributions of the d) power conversion efficiency (PCE), e) *V*_OC_, f) fill factor (FF), and g) *J*_SC_ values of the solar cells based on the SWCNT and TsOH‐SWCNT films.

The SWCNT films employed in this study were synthesized by the FCCVD method, and the as‐prepared films were directly transferred onto a target substrate using a simple method.^[^ ^9^, ^36^, ^37^ ^]^ Figure S1a (Supporting Information) indicates the procedure of preparing Si substrate with 3×3 mm^2^ active area surrounded with ≈300 nm thick SiO_x_, and the SEM image of the obtained substrate is shown in Figure S1b (Supporting Information). As‐prepared SWCNT film was transferred onto the Si substrate to form SWCNT/Si heterojunction (Figure S1c, Supporting Information). Subsequently, the TsOH precursor solution (**Figure** **1**a) was drop‐cast/spin‐coated to form the TsOH‐SWCNT/Si solar cell. Owing to the porous structure of the SWCNT film, the TsOH precursor solution reaches to the Si surface through the SWCNT network, resulting in building chemical bridges between SWCNTs and n‐type Si wafer (Figure 1b). Typical current density–voltage (*J‐V*) curves were measured under illumination with simulated AM 1.5G (100 mW cm^−2^) light, as shown in Figure 1c. The SWCNT/Si solar cell showed a *J*_SC_ of 29.8 mA cm^−2^, a *V*_OC_ of 0.582 V, an FF of 62.0%, and a PCE of 10.8%. After introducing TsOH, a champion PCE of 17.7%, with a *J*_SC_ of 35.5 mA cm^−2^, a *V*_OC_ of 0.623 V, and an FF of 80.0% were achieved for the TsOH‐SWCNT/Si device. Figure 1d–g shows statistical distribution of the photovoltaic parameters of 20 devices, we can see that both the SWCNT/Si and TsOH‐SWCNT/Si devices possess good reproducibility. The PCE improvement can be attributed to the simultaneously enhanced *J*_SC_, *V*_OC_, and FF.

**Figure 2:**
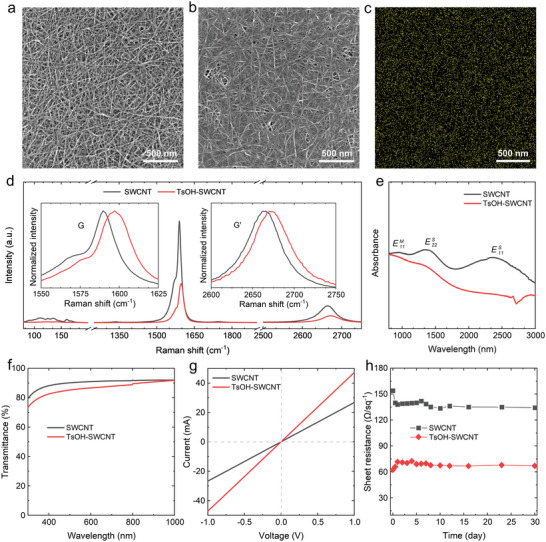
SEM images of the a) pristine single‐wall carbon nanotube (SWCNT) and b) p‐toluenesulfonic acid (TsOH) coated SWCNT (TsOH‐SWCNT) films on SiO_x_/Si substrates. c) Distribution of the S in the TsOH‐SWCNT film. d) Laser Raman spectra of the SWCNT and TsOH‐SWCNT films excited with 532 nm laser. e) Optical absorption, f) transmittance, g) *I‐V* curves, and h) long‐time stabilities of the SWCNT and TsOH‐SWCNT films.

To investigate the interaction between TsOH and SWCNT, the TsOH precursor solution was introduced in the SWCNT network. Figure S2 (Supporting Information) shows the TEM image of the pristine SWCNT film, which is composed of isolated and small‐bundled nanotubes with an average diameter of ≈2.1 nm. Typical SEM images (**Figure** **2**a and Figure S3a, Supporting Information) of the SWCNT film indicate a randomly oriented networked structure. Figure 2b and Figure S3b (Supporting Information) indicate SEM images of the TsOH coated SWCNT (TsOH‐SWCNT) film, it is found that TsOH uniformly covers the surface of the SWCNT network, which is further confirmed by the S elemental distribution shown in Figure 2c. The laser Raman spectra of the SWCNT films before and after TsOH coating are compared in Figure 2d. We can see that the pristine SWCNT film shows a high intensity ratio of G to D band (*I*_G_/*I*_D_ = 105), suggesting a high degree of graphitization. All the Raman peaks are dramatically suppressed after the TsOH coating, unexpectedly, the *I*_G_/*I*_D_ ratio is increased to 139.4, indicating that TsOH acts like a “patch” on the defects in the TsOH‐SWCNT film. In addition, blue shifts of G and G’ bands are clearly observed, which demonstrate the effective p‐type doping effect of TsOH for the SWCNT film.^[^ ^38^ ^]^ The optical absorption spectrum provides another route to investigate the properties of SWCNT films. Figure 2e shows optical absorption spectra of two samples, it is found that the first and second optical transitions of the van Hove singularities (*E*S 11 and *E*S 22) of semiconducting SWCNTs and the first optical transition of the van Hove singularities (*E*M 11) of metallic SWCNTs are dramatically quenched in the TsOH‐SWCNT film. This indicates that the Fermi level was downshifted owing to the charge transfer from SWCNTs to TsOH, resulting in the suppression of interband optical absorption.^[^ ^20^, ^39^ ^]^ To further confirm the p‐type doping effect of TsOH, the ultraviolet photoelectron spectroscopy (UPS) spectra of the SWCNT and TsOH‐SWCNT films are compared in Figure S4 (Supporting Information). The calculated work function of the pristine SWCNT film was 4.79 eV, while the work function of the TsOH‐SWCNT film was 5.02 eV, suggesting that the Fermi level was shifted due to the TsOH doping effect, which agrees with the results of Raman and optical absorption characterizations.

The optical transmittance spectra and current–voltage (*I‐V*) curves of the SWCNT film and TsOH‐SWCNT film were further measured. Figure 2f shows that the SWCNT film has high transmittance in the wavelength range of 300 to 1000 nm, and the transmittance is 90.5% for 550 nm light. The transmittance is slightly reduced after TsOH coating, which could be attributed to the extra light absorption by the TsOH layer (Figure S5, Supporting Information). The *I‐V* curves of the samples are compared in Figure 2g, suggesting that TsOH doping effectively enhanced the electrical conductivity of the SWCNT film. The influence of TsOH concentration on the sheet resistance of the SWCNT film is shown in Figure S6 (Supporting Information). It is found that its sheet resistance first decreases with the increase of TsOH concentration, and then stabilizes at a value of ≈62 Ω sq^−1^. In addition, we measured conductivity stabilities of these two simples in air for 1 month (Figure 2h). The sheet resistance of the pristine SWCNT film decreased to 134 from 154 Ω sq^−1^, which is attributed to the slight p‐type doping of oxygen molecules absorbed on the nanotube surface. This is also indicated by the decrease of the radial‐breathing mode (RBM) peak intensity and slight blue shift of G band for the SWCNT film exposed in air (Figure S7, Supporting Information). After 30 days in the ambient air, the sheet resistance of the TsOH‐SWCNT film slightly increased to 67 Ω sq^−1^. This result shows the excellent stability of the TsOH‐doped SWCNT film, demonstrating a promising potential of the TsOH‐SWCNT transparent conductive films.

**Figure 3:**
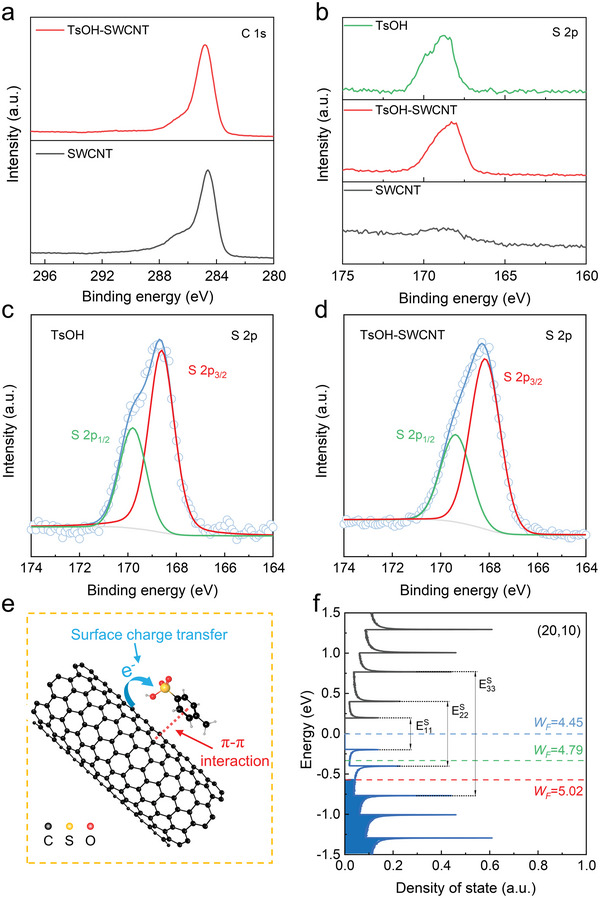
X‐ray photoelectron spectroscopy (XPS) spectra. a) C 1s and b) S 2p of the p‐toluenesulfonic acid (TsOH), single‐wall carbon nanotube (SWCNT) and TsOH‐SWCNT films. Gaussian curves fitting of S 2p peaks for c) TsOH and d) TsOH‐SWCNT films. e) Schematics of charge transfer in the TsOH‐SWCNT film. f) Density of state (DOS) of the (20, 10) semiconducting SWCNT with a diameter of ≈2.1 nm, its *E*S 11*, E*S 22*, E*S 33, and different work functions (*W*_F_) are also indicated.

X‐ray photoelectron spectroscopy (XPS) measurements were performed to investigate the interaction between TsOH molecules and SWCNTs. As we know, the C 1s spectrum of a SWCNT film is composed of five components (Figure S8a, Supporting Information), two of which are C=C (sp^2^) at ≈284.6 eV and C−C (sp^3^) at ≈285.6 eV. The C−C represents electron delocalization whose range may has been limited due to the chemical interaction and structure damage.^[^ ^40^ ^]^ In this work, the intensity ratio of C−C/C=C is 0.12 for the C 1s peak of the SWCNT film, which increases to 0.54 when the SWCNT film is coated by TsOH (Figure S8, Supporting Information). According to the Raman spectra shown in Figure 2d, there is no D peak detected in the TsOH‐SWCNT film, indicating that the structure of SWCNT is well retained. Therefore, the increase of the C−C/C=C intensity ratio in C 1s peak can be attributed to the interplay of TsOH with SWCNT sidewall.^[^ ^40^, ^41^ ^]^ In addition, we found that the width of the C 1s peak increased from 1.4 to 1.7 eV after the TsOH doping (**Figure** **3**a), which may be caused by the limited electron delocalization due to the *π*–*π* interaction between TsOH and SWCNTs.^[^ ^42^, ^43^ ^]^ Thus, the new peak located at 291 eV in Figure S8b (Supporting Information) can be ascribe to the *π*–*π* interaction, which agrees with previous studies.^[^ ^44^ ^]^ Apart from XPS results, absorbance spectra could confirm the existence of this interplay. As shown in Figure S5 (Supporting Information), the initial SWCNT film is characterized by an optical absorption peak at 267 nm due to the *π* plasmon, which represents the collective excitation of the *π*‐electron system.^[^ ^45^, ^46^ ^]^ The presence and interaction of TsOH on the SWCNT film is validated by the occurrence of a new peak at 223 nm and the shift and enhancement of *π*‐plasmon peak, respectively. These results suggest that the TsOH is adsorbed on the surface of SWCNT via a *π*–*π* stacking, which contributes to the charge transfer between SWCNTs and TsOH molecules.

The charge transfer between the TsOH molecule and SWCNT film is also revealed by the XPS analysis. Figure 3b shows S 2p spectra of the TsOH, SWCNT, and TsOH‐SWCNT. A very weak S 2p peak is observed for the SWCNT film, which can be attributed to the residual sulfur growth promoter used during the FCCVD synthesis process. The S 2p peak of the TsOH‐SWCNT film shifts to lower binding energy compared to that of TsOH. Through Gaussian curve fitting of the S 2p peaks of TsOH (Figure 3c), S 2p_3/2_ and S 2p_1/2_ are observed at 168.6 and 169.8 eV, respectively. While for the TsOH‐SWCNT film, the S 2p_3/2_ and S 2p_1/2_ locate at 168.3 and 169.5 eV, respectively (Figure 3d), and both shifted 0.3 eV toward lower binding energy. In addition, the O 1s peak of the TsOH‐SWCNT film shifts to lower binding energy compared to that of TsOH (Figure S9, Supporting Information). These results originated from the strong electron‐withdrawing property of the sulphonic group, ^[^ ^47^, ^48^ ^]^ leading to the transferred electrons from SWCNTs to TsOH dopants. A schematic showing the charge transfer in TsOH‐SWCNT films is illustrated in Figure 3e. The *π*–*π* interaction between the TsOH and SWCNTs is helpful for their contact and the sulphonic group can accept the charge from SWCNTs, resulting in an effective p‐type doping and the Fermi level of the SWCNT film shifts downward to the valence band. According to the previous reports,^[^ ^17^ ^]^ the work function of a SWCNT film without absorbing oxygen was about 4.45 eV. Owing to the oxygen doping in air, the work function increased to 4.79 eV. After TsOH doping, the work function was further improved to 5.02 eV (Figure S4, Supporting Information). The average diameter of SWCNTs prepared in our work is ≈2.1 nm, which can be calculated from the position of RBM peak shown in Figure 2d according to the formula of *d* = 248/*δ*_RBM_.^[^ ^49^ ^]^ For a (20, 10) semiconducting SWCNT with a diameter of 2.1 nm, its density of states (DOS) can be calculated by the tight binding model,^[^ ^50^ ^]^ as shown in Figure 3f. The Fermi level shifts to the location between *E*S 11 and *E*S 22 due to the influence of environmental oxygen.^[^ ^51^ ^]^ After introducing TsOH, the Fermi level was further downshifted, and its position was between *E*S 22 and *E*S 33, which agrees with the significant suppressions of the first and second optical transition of *E*S 11 and *E*S 22 (Figure 2e). Thus, the TsOH works as an efficient p‐type dopant to tune the work function of SWCNT films.

**Figure 4:**
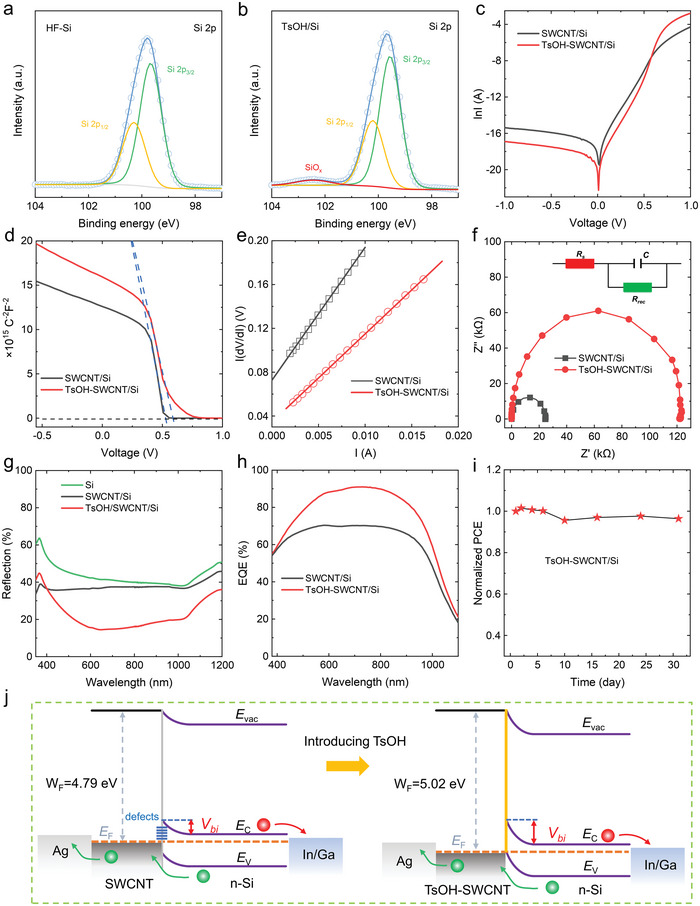
Gaussian curves fitting of Si 2p peaks for a) the HF‐treated Si and b) the p‐toluenesulfonic acid (TsOH)/Si. c) Dark *J‐V* curves, d) 1/*C* ^2^‐*V* plots, e) *I* (d*V*/d*I*) as a function of I, f) Electrochemical impedance spectroscopy (EIS) data, g) reflectance spectra, and h) external quantum efficiency (EQE) spectra of the single‐wall carbon nanotube/silicon (SWCNT/Si) and TsOH‐SWCNT/Si solar cells. i) Power conversion efficiency (PCE) stability of the TsOH‐SWCNT/Si solar cell put in air over 1 month. j) Schematic diagrams showing simplified energy bands of devices without (left) and with (right) TsOH.

To confirm the interaction between TsOH and Si wafer, the surface structure of HF‐treated Si and TsOH/Si were characterized using XPS spectra (Figure S10, Supporting Information), and Gaussian fittings of the curves are shown in **Figure** **4**a,b. We can see that the Si 2p peak of the HF‐treated Si locates at 99.9 eV, which can be attributed to Si–Si bonds, and there is no SiO_x_ peak observed at higher binding energy. While for the TsOH/Si wafer, the Si 2p peak is centered at 99.7 eV, lower than that of the HF‐treated Si. The decrease of the Si 2p binding energy is helpful for improving the surface quality and forming surface band bending in Si, which is similar to n‐Si coated with organic molecules.^[^ ^34^, ^52^, ^53^ ^]^ Interestingly, a Si 2p peak is detected in the range of 102–103 eV, which can be ascribed to the Si–O bond,^[^ ^34^, ^53^ ^]^ suggesting the presence of Si sub‐oxide on the TsOH/Si surface.^[^ ^32^, ^54^ ^]^ This result indicates that the oxygen in the sulfonic functional group in the TsOH molecule grafts on the dangling bonds of Si substrate to generate SiO_x_, which agrees with previous reports.^[^ ^32^, ^33^ ^]^ Therefore, the coated TsOH passivates the surface of the n‐type Si, leading to suppressed charge recombination in Si‐based solar cells.

To reveal the high *V*_OC_ achieved in the TsOH‐SWCNT/Si devices, dark *J*–*V* characteristics were measured. The relationship between *I* and *V* is described below,

(1) $$I\;=I_{0}\;\left[\exp\left(\frac{qV}{nKT}\right)-1\right]\;$$

where *I*_0_ is the reverse saturation current, *q* is the electronic charge, *V* is the applied voltage, *n* is the diode ideality factor, *K* is the Boltzmann constant, and *T* is the temperature. Figure 4c shows the ln*I*–*V* curves for two devices. After introducing TsOH, the value of *n* reduces from 2.1 to 1.2, which suggests that the junction quality is dramatically improved and the carrier recombination at the SWCNT/Si interface is suppressed, leading to a higher *V*_OC_. Moreover, based on the relationship between *V*_OC_ and *I*_0_ demonstrated below,

(2) $$V_{\mathrm{OC}}=\frac{KT}{q}\;ln\frac{I_{\mathrm{SC}}}{I_{0}}+1\;$$

we can see that the reducement of *I*_0_ is helpful for enhancing *V*_OC_. The calculated value of *I*_0_ is decreased from 1.25 × 10^−4^ mA cm^−2^ to 2.81 × 10^−7^ mA cm^−2^ when the device is coated with TsOH, which accounts for an increase in the value of *V*_OC_. Capacitance–voltage (*C−V*) measurements were adopted to investigate the capacitance variation of these two devices under bias voltage and the curves of 1/*C* ^2^−*V* are shown in Figure 4d. The built‐in potential (*V*_bi_) can be extracted from the slope acording to:

(3) $$\frac{1}{C^{2}}=\frac{2\left(V_{\mathrm{bi}}-V\right)}{q\varepsilon N_{D}}$$

where *ε* and *N*_D_ are the permittivity and doping level of Si, respectively. The value of *V*_bi_ increased by ≈0.045 V, which is accorded with the enahcement of *V*_OC_. Therefore, higher *V*_OC_ of the TsOH‐SWCNT/Si device can be obtained by improving junction quality and the separation capabilities of carriers.

Figure 4e shows linear fitting of *I* (d*V*/d*I*) as a function of *I* for two solar cells. The series resistances (*R*_S_) can be calcultated from the slopes, which suggest that the *R*_S_ values of the SWCNT/Si and TsOH‐SWCNT/Si devices are 12.1 and 8.0 Ω, respectively. The decrease of *R*_S_ originates from the lower sheet resistance of the SWCNT film doped with TsOH, which efficiently enhances charge transport and leads to higher *J*_SC_ and FF. Electrochemical impedance spectroscopy was measured under dark conditions, and the Nyquist plots are shown in Figure 4f. For both solar cells, the curves are near semicircle in shape, indicating that only a single junction exists at the device interface.^[^ ^35^ ^]^ The TsOH‐SWCNT/Si device exhibits a much higher carrier recombination resistance (*R*_P_) than the SWCNT/Si device, as indicated by the larger diameter for the corresponding semicircle. This suggests that charge recombination is effectively suppressed owing to the passivation effect of TsOH, which is consistent with the observed significantly increased FF value. In addition, the lower *n* reveals a higher quality heterojunction formed in the TsOH‐SWCNT/Si device (Figure 4c), which contributes to the reduced trap density and/or enhanced extraction of the photogenerated carriers at the interface.

To clarify the origin of the significantly improved *J*_SC_ in the existence of TsOH layer, we measured the reflectance spectra and external quantum efficiency (EQE) of the SWCNT/Si and TsOH‐SWCNT/Si solar cells. As shown in Figure 4g, the average reflectance of a planar Si substrate is over 38% in the wavelength range of 350 to 1100 nm owing to its high refractive index. After transfering a SWCNT film, the reflectivity is slightly reduced, which may be attributed to the light absorption of SWCNTs. The TsOH coating dramatically decreases the light reflection in the spectral range of 400 to 1100 nm, especially at 650 nm, the reflection is as low as 14%. This result shows that the TsOH layer acts as an effective antireflective film in Si‐based solar cells. Moreover, the optical image of the SWCNT/Si heterojunction shows a gray color, while it changes to a deep blue color after the coating of TsOH layer (Figure S11, Supporting Information). The reflection spectra of the TsOH coating with different thicknesses are displayed in Figure S12a (Supporting Information). It can be seen that the lowest reflectivity shifts toward higher wavelength when increasing the concentration of the TsOH solution, suggesting that this antireflection effect originates from the destructive interference due to the different refractive indices between the TsOH layer and the Si wafer (Figure S12b, Supporting Information). The antiflection effect of the TsOH layer is beneficial for improving light absorption by the device, leading to an enhanced *J*_SC_. The EQE spectra of the two devices are compared in Figure 4h. For the TsOH‐SWCNT/Si device, the EQE values in the range of 400 to 1000 nm reach ≈90% and are higher than that of the SWCNT/Si solar cell, which agrees well with the reduced reflectivity in the same spectral range. The photocurrent densities calculated from EQE spectra for the SWNCT/Si and TsOH‐SWCNT/Si devices are 28.6 and 35.4 mA cm^−2^, respectively, which are very close to the *J*_SC_ values measured from the *J*–*V* curves (Figure 1c). Therefore, the reduced reflectivity and enhanced EQE cause a significant increase in *J*_SC_ after introducing TsOH layer. Finally, we evaluate the stability of the TsOH‐SWCNT/Si solar cell by exposure in air, the PCE retains over 96% of its initial value after 1 month (Figure 4i). The key of the good stability of the TsOH‐SWCNT/Si solar cell lies in the stable doping from the solid TsOH.

Figure 4j shows energy band diagrams of SWCNT/Si devices before and after introducing TsOH. Based on the Schottky theory, the barrier height (*V*_bi_) of the heterojunction between SWCNT and Si is determined by the difference of their work function. The TsOH‐SWCNT film possesses a lower Fermi level than that of the SWCNT film, resulting in a higher barrier height and larger photovoltage. In addition, interfacial defects at the SWCNT/Si heterojunction can be effectively reduced by the TsOH molecules. Based on the above analyses, it is found that TsOH can work as multifunctional roles in SWCNT/Si heterojunction solar cells. Briefly, TsOH is absorbed on the surface of SWCNTs through *π*–*π* interaction, and the sulphonic group withdraw electrons from SWCNTs, resulting in a p‐type doping of the SWCNT film. Meanwhile, the oxygen in the sulfonic functional group in the TsOH molecule grafts on the dangling bonds of the Si surface, causing a passivation effect. These two factors cause significantly improved *V*_OC_ and FF. Moreover, the TsOH coating functions as an antireflective coating to enhance light absorption by solar cells, leading to a measurable increment in *J*_SC_. Therefore, the promoted photovoltaic performance of TsOH‐SWCNT/Si solar cells can be attributed to the multifunction of TsOH layer, including antireflection, doping effect, chemical bridge, and defect passivation. Although the TsOH dopants can effectively reduce the sheet resistance of the SWCNT film, its performance is still not as good as ITO electrodes. In future works, highly performance, stable, and economical SWCNT films need to be developed to further boost the performance of the SWCNT/Si heterojunction solar cells.

## 3 Conclusions

We have successfully applied TsOH as a multifunctional layer in SWCNT/Si heterojunction solar cells. The conductivity and work function of SWCNT films were efficiently enhanced and tuned due to the surface charge transfer doping of TsOH. Meanwhile, SWCNTs and the phenyl group of TsOH are chemically coupled through *π*–*π* interaction. In addition, the oxygen in the sulfonic functional group of TsOH grafts on the Si surface, leading to a passivation effect. As a result, a chemical bridge structure is successfully built at the SWCNT/Si interface, which significantly improved FF and *V*_OC_. Owing to the antireflection effect of the TsOH coating layer, an improved *J*_SC_ was obtained. Finally, a high PCE value of 17.7% was achieved for the TsOH‐SWCNT/Si device, and it exhibited outstanding stability in atmosphere environment. Our work presents a new avenue toward fabricating efficient, stable, and reproducible carbon nanotube/silicon photovoltaic devices.

## 4 Experimental Section

### Preparation of SWCNT Films and TsOH‐SWCNT Films

SWCNT films were synthesized by a FCCVD method, as reported in the previous works.^[^ ^8^, ^9^ ^]^ Ethylene and hydrogen were used as the carbon source and carrier gas, respectively. A mixed solution containing toluene, ferrocene, and thiophene was employed as the liquid carbon source, catalyst precursor, and growth promoter, and was injected into a quartz tube reactor by a syringe pump. The growth temperature was 1100 °C. The SWCNT film was collected on a membrane filter (0.45 µm pore diameter) at room temperature and could be easily transferred onto a target substrate. The solid p‐toluenesulfonic acid was dispersed in isopropanol with different concentrations to prepare dopant precursors, which were spin‐coated on SWCNT films to form TsOH‐SWCNT films.

### Fabrication of Heterojunction Solar Cells

A n‐type single‐crystalline silicon wafer (the bulk resistivity is 2–4 Ω cm) covered with a 300 nm‐thick thermal silicon oxide was patterned with a square window (≈3 × 3 mm), and the silicon oxide in the square area was then etched away by a buffered oxide etchant (6:1 of 40% NH_4_F and 49% HF) and rinsed with deionized water and isopropanol to form the active area (≈0.09 cm^2^). The SWCNT film was transferred onto the top surface of the Si substrate to fabricate the SWCNT/Si heterojunction. Micrometer‐thick silver paste was spread around the active area to work as a front electrode, while an ohmic contact at the back of the silicon wafer was formed by using liquid‐state gallium‐indium eutectic. The TsOH‐SWCNT/Si devices could be achieved by introducing the TsOH solution in SWCNT/Si device using a spin‐coated technique.

### Characterization

The structure and morphology of films was characterized by SEM (Nova Nano SEM 430) and TEM (Tecnai F20, operated at 200 kV). Raman spectra of films were obtained using a Jobin‐Yvon Labram HR800 instrument, excited with 532 and 633 nm lasers. XPS spectra were measured using the Thermo Scientific K‐Alpha. UPS spectra were conducted by Thermo 250Xi and Thermo Nexsa G2. The optical absorption and transmission spectra of the films and the reflectance spectra of the solar cells were measured using an UV–Vis–NIR spectrophotometer (AGILENT CARY 5000) equipped with an integration sphere. The *I*–*V* curves of the films were measured by a source meter (Keithley 2400). Solar cell characteristics were determined by a solar simulator under AM 1.5 G (100 mW cm^−2^) light and a source meter (Keithley 2400). The irradiation intensity was calibrated using a standard Si solar cell. EIS measurements at a frequency range 10 Hz to 1 MHz and *C*–*V* measurement was swept from −1 to 1 V under dark conditions by an electrochemical workstation (BioLogic VSP‐300).

## Conflict of Interest

The authors declare no conflict of interest.

## Supporting information

Supporting Information

## Data Availability

The data that support the findings of this study are available from the corresponding author upon reasonable request.
