# Supplementary material for: Interfacial Chemical Bridging Constructed by Multifunctional Lewis Acid for Carbon Nanotube/Silicon Heterojunction Solar Cells with an Efficiency Approaching 17.7%
Source: Adv Sci (Weinh). 2023 Feb 23;10(13):2206989. doi: 10.1002/advs.202206989 (PMC10161097; doi:10.1002/advs.202206989)
Supplement: Supplementary file 1 — Supporting Information [file ADVS-10-2206989-s001.pdf]

ADVANCED SCIENCE
Open Access

Supporting Information

for *Adv. Sci.*, DOI 10.1002/advs.202206989

Interfacial Chemical Bridging Constructed by Multifunctional Lewis Acid for Carbon Nanotube/Silicon Heterojunction Solar Cells with an Efficiency Approaching 17.7%

Xian-Gang Hu, Yi-Ming Zhao, Hongyu Yang, Peng-Xiang Hou, Chang Liu, Jingjing Chang* and Yue Hao*

Supporting Information

Interfacial chemical bridging constructed by multifunctional Lewis acid for carbon nanotube/silicon heterojunction solar cells with an efficiency approaching 17.7%

Xian-Gang Hu[a] Yi-Ming Zhao[c], Hongyu Yang[a], Peng-Xiang Hou[c], Chang Liu[c*], Jingjing Chang[a,b*], Yue Hao[a,b]

a Advanced Interdisciplinary Research Center for Flexible Electronics, Academy of Advanced Interdisciplinary Research, Xidian University, Xi'an 710071, P. R. China

b State Key Discipline Laboratory of Wide Band Gap Semiconductor Technology, School of Microelectronics, Xidian University, Xi'an 710071, China

c Shenyang National Laboratory for Materials Science, Institute of Metal Research, Chinese Academy of Sciences, Shenyang, 110016, China

Corresponding Authors: cliu@imr.ac.cn; jjingchang@xidian.edu.cn;

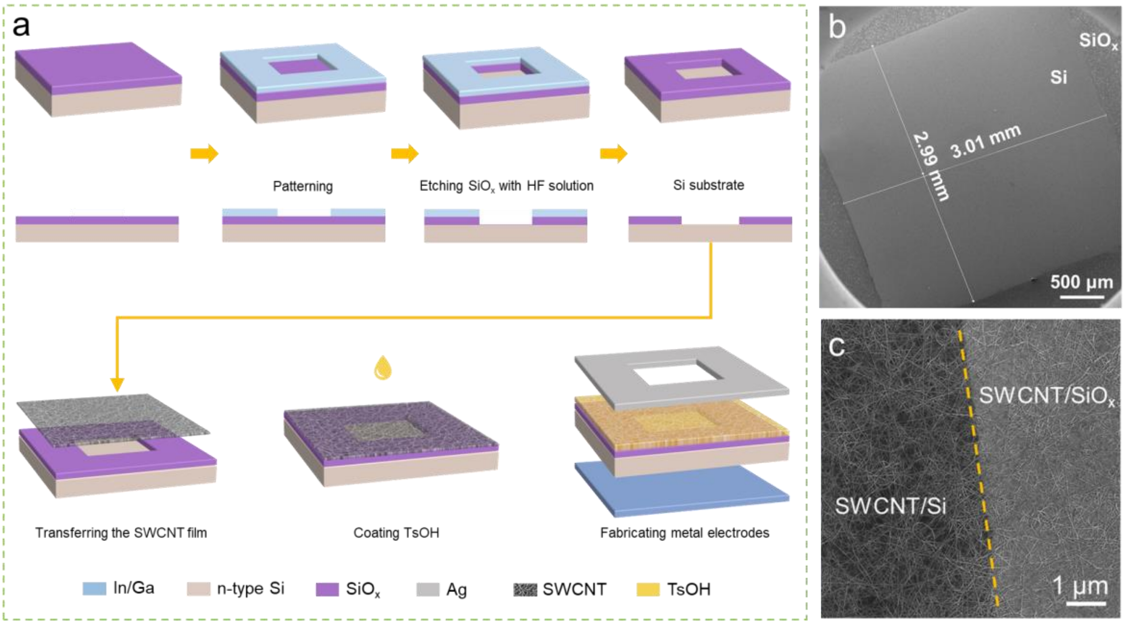

Figure S1. a) The procedure of the TsOH-SWCNT/Si device preparation. b) SEM image of a Si substrate with 9 mm^2 active area surrounded by ~300 nm thick SiO_x. c) SEM image of a SWCNT film on the Si/ SiO_x substrate.

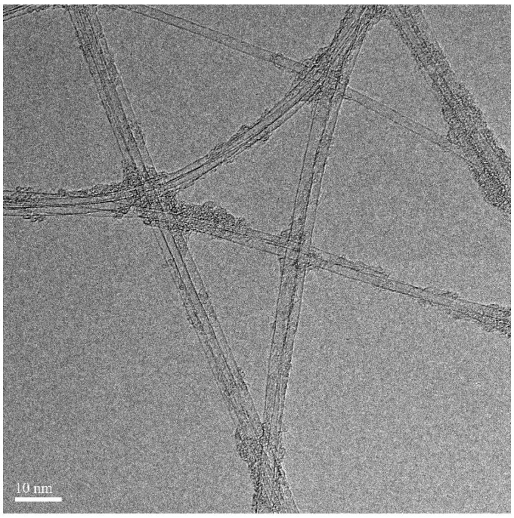

Figure S2. Typical TEM image of the small-bundled SWCNTs.

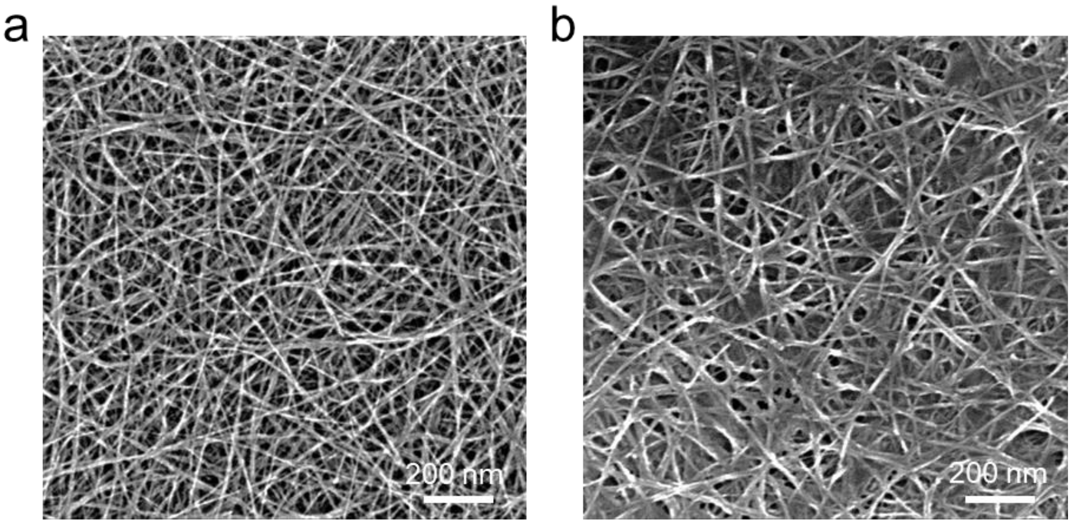

Figure S3. High-magnification SEM images of the a) SWCNT film and b) TsOH-SWCNT film.

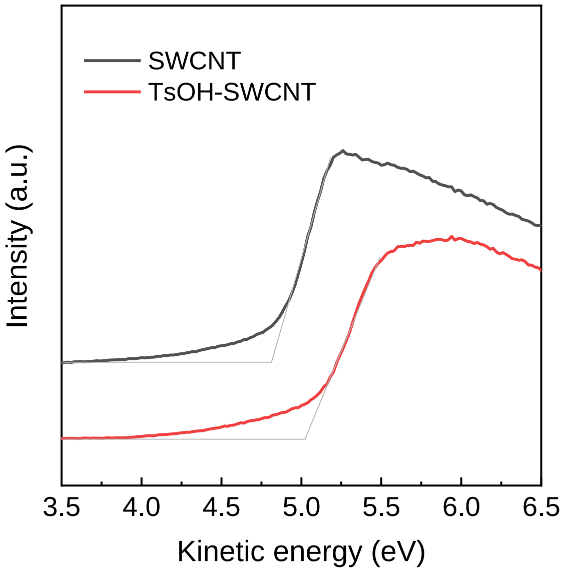

Figure S4. UPS spectra of the SWCNT and TsOH-SWCNT films.

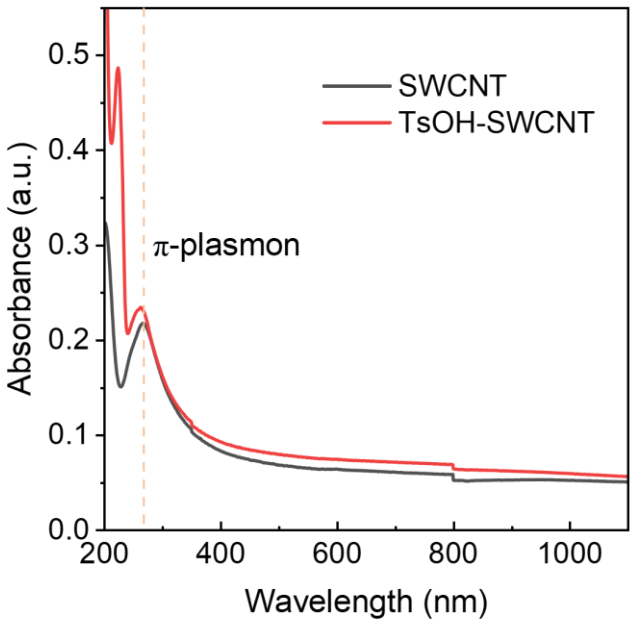

Figure S5. Optical absorption spectra of the SWCNT and TsOH-SWCNT films at the wavelength range of 300~1100 nm.

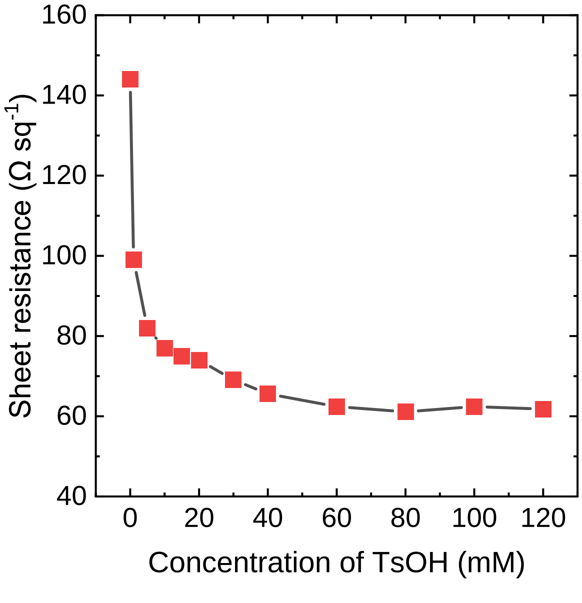

Figure S6. The influence of TsOH concentration on the sheet resistance of SWCNT films.

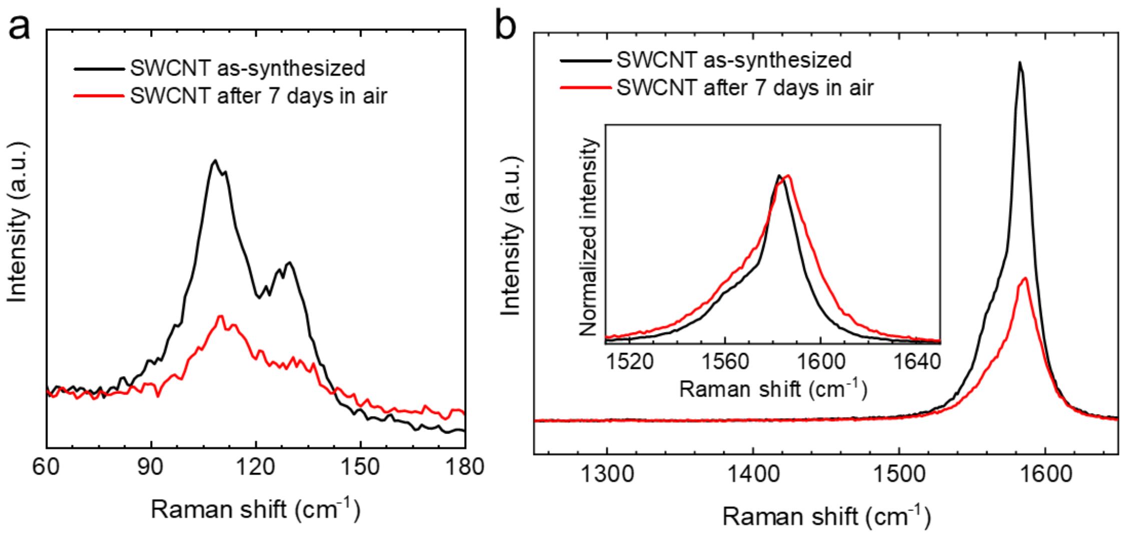

Figure S7. Raman spectra of as-prepared SWCNT films and that exposed in air for a week. a) RBM and b) G band.

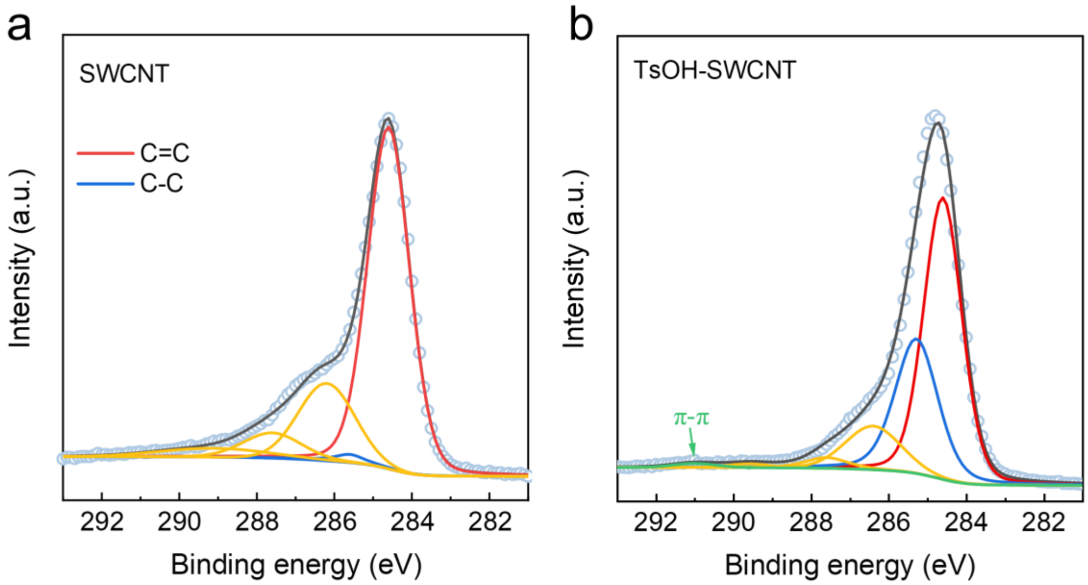

Figure S8. The C 1s spectra of the SWCNT and TsOH-SWCNT films.

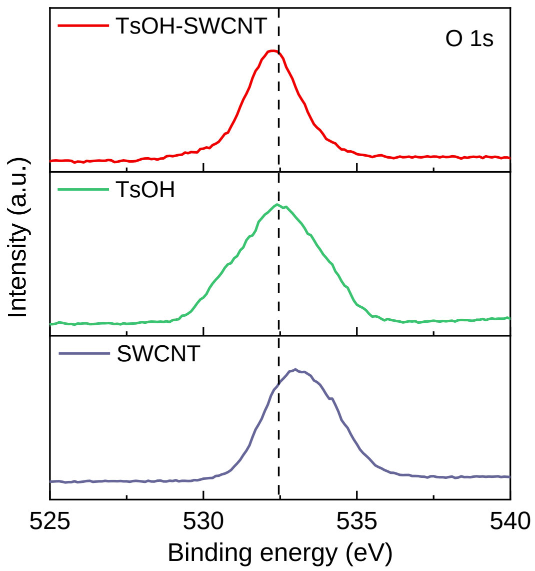

Figure S9. O 1s spectra of the TsOH, SWCNT and TsOH-SWCNT films.

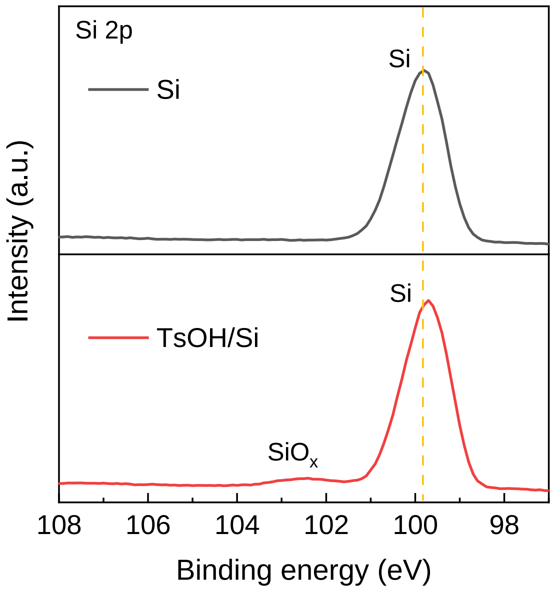

Figure S10. Si 2p spectra of the SWCNT and TsOH-SWCNT films.

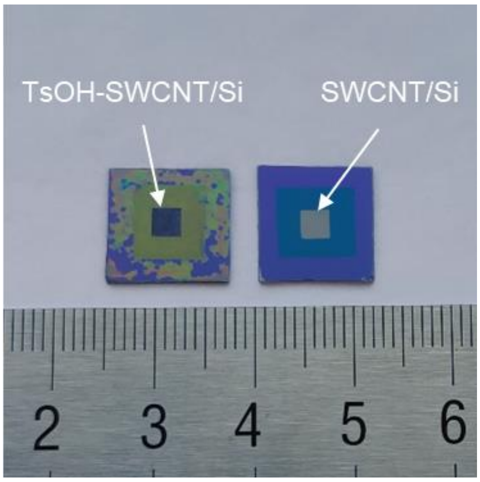

Figure S11. Optical images of the SWCNT/Si and TsOH-SWCNT/Si devices.

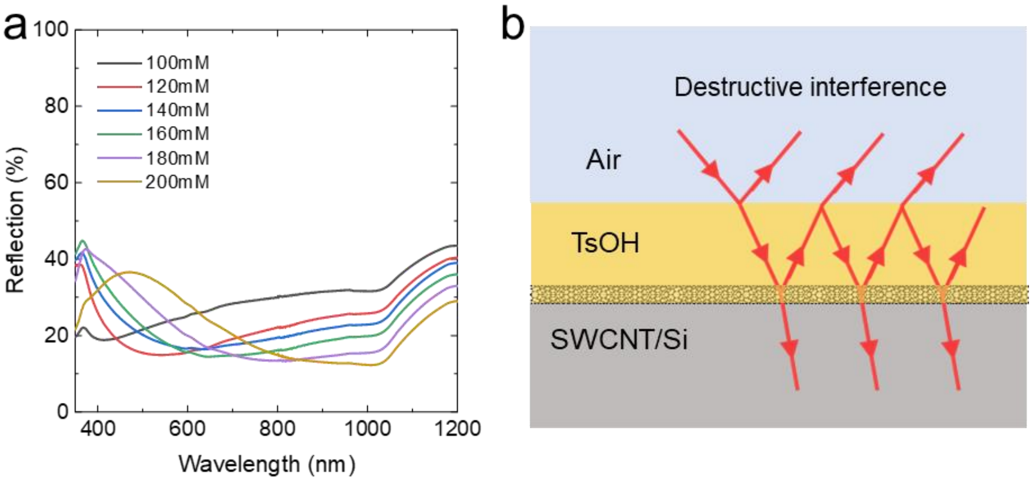

Figure S12. a) The reflection spectra of the TsOH coating with different thickness on SWCNT/Si devices. b) Schematic diagram indicating the antireflection effect of the TsOH layer.
